# Abnormal brain network reconfiguration in neuropsychiatric disorders across cognitive decline, Depression, and Schizophrenia

**DOI:** 10.1371/journal.pone.0337470

**Published:** 2025-12-04

**Authors:** Yan He, Zhiqiang Yan, Yuan Liang, Yunli Yu

**Affiliations:** 1 School of Tourism, School of Artificial Intelligence, Xi’an International Studies University, Xi’an, China; 2 Department of Neurosurgery, Xijing Hosptial, The Fourth Military Medical University, Xi’an, China; 3 Key Laboratory for Artificial Intelligence and Cognitive Neuroscience of Language, Xi’an International Studies University, Xi’an, China; 4 Department of Neurology, The Affiliated Hospital of Guizhou Medical University, Guizhou Medical University, Guiyang, China; Rutgers University Newark, UNITED STATES OF AMERICA

## Abstract

**Objective:**

Neuropsychiatric disorders are characterized by high complexity and comorbidity, imposing a substantial burden on both patients and society. However, their elusive pathogenic mechanisms impede accurate clinical diagnosis and effective interventions. To overcome this challenge, the present study proposes a novel framework to quantify and characterize these disorders.

**Methods:**

Routine electroencephalogram (EEG) recordings are acquired from 236 subjects, including patients with Alzheimer’s disease (AD), mild cognitive impairment (MCI), major depressive disorder (MDD), schizophrenia, and healthy controls (HCs). Time-varying functional brain networks are constructed by phase locking value (PLV) analysis on band-pass filtered EEG signals. Subsequently, the nodal behavior characteristics within these dynamic brain networks are quantified by integrating robust dynamic community detection algorithms and network reconfiguration metrics.

**Results:**

Significant intergroup differences in network reconfiguration metrics are identified based on the dynamic community structures (FDR-corrected *p < 0.001*). Lower cohesion strength is observed across all neuropsychiatric disorders compared to healthy controls, consistent across all frequency bands and recording sites. When six machine learning classifiers are trained on these metrics, the maximum classification accuracies exceeded 80%. Since lower cohesion strength is a prominent potential biomarker for neuropsychiatric disorders, it was then selected as the independent input feature for random forest classifier, and the classification accuracy achieved 0.85 for schizophrenia group, 0.88 for both the MCI and MDD group, and 0.82 for the AD group.

**Conclusions:**

Our findings indicate that the framework based on dynamic network reconfiguration metrics effectively captures both the shared and disorder-specific alterations in brain network dynamics among neuropsychiatric disorders.

**Significance:**

Dynamic community structure advances our understanding of the pathological mechanisms underlying neuropsychiatric disorders. This study provides novel insights that may inform the development of more targeted and effective therapeutic strategies.

## 1. Introduction

Depression, schizophrenia, and cognitive decline represent major global public health challenges. Recent research has increasingly emphasized the interrelated nature of these neuropsychiatric disorders. Cognitive impairment is a core feature of schizophrenia, manifesting as disruptions in thought, perception, and emotion [1–3]. In particular, cognitive dysfunction in schizophrenia impairs executive function, social functioning and overall quality of life [1,3]. Elucidating neuropsychological functioning in schizophrenia is thus essential for understanding its cognitive dynamics [2]. Patients with Alzheimer’s disease (AD) and comorbid depression experience accelerated cognitive decline, which is partly attributable to frontal atrophy [4]. In addition, mild cognitive impairment (MCI) is associated with an elevated risk of subsequent depression [5]. Conversely, depression can increase cognitive dysfunction and accelerate decline [6–8]. Depression in midlife may elevate the risk of developing AD later in life, whereas late-life depression may serve as a precursor or early manifestation of dementia [9]. Moreover, apathy, mood dysregulation, and impulse control difficulties are frequently observed in the early stages of MCI [10–11]Shared biological pathways underlying depression and cognitive dysfunction may include neural inflammation, cardiometabolic dysregulation, dopaminergic neurotransmission abnormalities, and insufficient social support systems [11–12]. Notably, amyloid-beta (Aβ) accumulation occurs persistently in patients with both MCI and major depressive disorder patients. Emerging evidence indicates that lipopolysaccharide-induced immune activation may aggravate Aβ deposition, thereby worsening cognitive decline and depressive symptoms [13]. Neuropsychiatric disorders such as AD and schizophrenia pose substantial clinical challenges owning to their complexity and high rates of comorbidity. Current diagnostic approaches rely predominantly on subjective assessments, which often fail to predict disease progression or treatment response accurately [14]. A systematic investigation of distinctive neurophysiological features in these disorders could illuminate their underlying mechanisms and improve diagnostic precision and treatment efficacy. Overall, the complex interplay among depression, AD, and other neuropsychiatric conditions remains elusive and warrants further objective investigation.

Neural oscillations serve as critical biomarkers of cognitive function and neuropsychiatric disorders. Advanced techniques for recording and decoding brain activity offer powerful tools for elucidating the mechanisms of these conditions [15–18]. Physiological studies reveal that brain oscillations across different frequency bands and timescales play distinct roles in action preparation, execution, and feedback processing [19–20]. Remarkably, olfactory task paradigms demonstrate a substantial reduction in the prefrontal alpha and theta band activity in MCI and AD patients. These oscillatory abnormalities reflect deficits in olfactory processing and cognitive integration in AD and MCI, demonstrating strong correlations with scores on cognitive assessment such as the Mini-Mental State Examination (MMSE) [21]. Furthermore, patients with AD exhibit reduced neural synchronization between the hippocampal and prefrontal cortical regions in the alpha and beta frequency bands [22–23].

Over the past several centuries, the human brain has been conceptualized as a complex network system, wherein neuronal populations organize into distinct architectures through anatomical wiring and functional coupling [12,24–29]n. Advances in neuroimaging firmly established that large-scale integration and interregional cooperation are essential for sustaining cognitive functions.

In the present study, a brain network is defined as a complex system comprising nodes and edges, where nodes correspond to recording sites (a coarse-grained representation of brain regions), and the edges denote the statistical dependence between those nodes. The healthy brain exhibits a characteristic small-world architecture, which reconciles the competing demands of global integration and local segregation of neural behaviors.

Connectome fingerprinting has revealed that individual functional connectivity profiles are unique and stable over sessions and scanners, enabling robust subject identification [24]. Cross-diagnostic investigations further demonstrate that aberrant hippocampal enlargement represents a shared substrate underlie disparate psychiatric phenotypes [12]. Moreover, dysregulation of the default mode network emerges as a convergent functional motif across depression, anxiety and schizophrenia [29].

Collectively, brain network analysis provides innovative avenues for cross-diagnostic taxonomic classification and the identification of disease-specific biomarkers. Alterations in brain network architecture serve as sensitive biomarkers for the early detection and clinical monitoring of AD [22,23,30]. In patients with MCI, depressive symptoms are associated with both reduced gray matter volume and attenuated network function, which are predictive of subsequent cognitive decline and progression to AD [4,31]. Late-life depression is marked by elevated posterior gamma power and disrupted theta-band connectivity between parietal and fronto-occipital regions, reflecting underlying impairments in attention and executive control [32].

Schizophrenia is characterized by distinct static network abnormalities, including impaired functional connectivity [33–34] and disrupted synchronization between structural and functional networks [35]. Similarly, major depressive disorder (MDD) demonstrates disintegration of intrinsic functional networks, as evidenced by altered connectivity patterns, nodal centrality metrics, and aberrant modular organization when compared to healthy controls [36]. These disruptions are further marked by reduced global efficiency, increased modularity and attenuated small-world properties [37–38]. The integrated investigation of neuropsychiatric disorders, including mood and anxiety disorders, combined with analysis of large-scale rapid temporal network dynamics, yields critical insights into both normal neural network function and pathological circuit mechanisms [39–40].

Temporal coordination and dynamic network properties are fundamental to comprehending neurological disorders. Data-driven dynamic functional connectivity is an effective biomarker for identifying brain disorders [39–40]. Early signs of dementia progression can be detected through shortened microstate durations, reduced transition probability, and decreased network coverage that indicative of cortical degradation in patients with MCI [41]. Cognitive decline in MCI is further associated with altered coherence in theta and alpha frequency bands [16] and diminished dynamic integration during prospective memory tasks [42]. MDD demonstrates abnormal cross-frequency coupling between low (delta, theta) and high (beta, gamma) frequency bands, suggesting impaired inter-band integration [37]. In schizophrenia, dynamic network abnormalities manifest as inflexible reconfiguration patterns, particularly in theta and gamma bands, which are critical for cognitive processing. This rigidity may contribute to the observed cognitive deficits and attentional impairments [43].

Recent methodological advances hold considerable promise for enhancing diagnosis and longitudinal monitoring. Self-attention mechanisms facilitate more effective feature extraction from EEG signals in AD by applying different temporal weighting across time points [44]. Spatio-temporal pattern recognition in brain networks achieves high classification accuracy on diverse neuropsychiatric cohorts [38]. These findings underscore the pivotal role of temporal coordination in cortical networks and its potential in elucidating core mechanisms of brain disorders [19,45].

Community detection algorithms effectively differentiate patients with schizophrenia from healthy controls by identifying distinctive interaction patterns among brain regions [46]. Unlike global metrics such as clustering coefficient or path length, a community is defined as a subset of nodes that assemble more compact than others, thereby representing cohesive subnetworks within the broader brain system. Recent advances combining community structure analysis with graph convolutional networks have enhanced the identification of disease-affected regions and connections [47]. In schizophrenia, altered community structure specifically reflects network perturbations associated with stress-response pathways [48]. Comparable approaches reveal diminished community dynamics appears in MDD relative to healthy controls, with pronounced differences in the visual and default mode network across all frequency bands, along with the cognitive control network in alpha-2 and beta bands, and the bilateral limbic network in beta band, as derived from magnetoencephalogram signals (Zhong et al., 2023). These findings suggest that cognitive impairment in neuropsychiatric disorders may arise from aberrant community structure, where dysregulated neurodynamics disrupt brain function across multiple spatiotemporal scales [43,45,49,50].

Despite substantial progress, significant challenges persist in neuropsychiatric research owing to incomplete mechanistic understanding and barriers to clinical translation. While neuroimaging-based brain network analysis partly informs precision therapeutics, specific brain network patterns have been identified as the core neural substrates of these disorders, where traditional static connectivity methods would fail to capture due to their inability to resolve rapid fluctuations in neural oscillations [51–53]. With its superior temporal resolution, EEG is particularly suitable for digging into these dynamic network characteristics that correlate with symptom severity and underlying pathophysiology [54]. Systematic investigation of such network aberrations may unravel the biological foundation of neuropsychiatric disorders and inform future therapeutic development.

Accordingly, the present study employs dynamic community detection and network reconfiguration analysis to characterize time-varying functional networks across MCI, Alzheimer’s disease (AD), major depressive disorder (MDD), and schizophrenia. The proposed framework identifies altered community evolution and reduced temporal cohesion as two cardinal pathological features common to these disorders. To evaluate the discriminative power of these network reconfiguration metrics, six machine learning classifiers are implemented. Comparative analyses demonstrate that Support Vector Machine (SVM), k-Nearest Neighbors (KNN) and random forest (RF) outperform other algorithms in pairwise group discrimination, achieving cross-validated accuracies exceeding 80% for each clinical cohort. These results establish network reconfiguration metrics as promising neural biomarkers. By integrating multilayer modularity optimization with temporal cohesion-disjointedness dynamics, this investigation offers novel mechanistic insights into the pathophysiology of neuropsychiatric disorders.

## 2. Methods and materials

### 2.1 Participants

This study included four neuropsychiatric disorders with publicly available EEG data [55]. The research was conducted in accordance with the Declaration of Helsinki and approved by the Institutional Review Board (IRB) of the Rabin Medical Center, Petach Tikva, Israel (0275-20-RMC). All participants provided written informed consent, and data were fully anonymized prior to analysis [55]. In addition to 96 healthy controls, 43 participants were diagnosed with Alzheimer’s disease (AD), 27 with a primary diagnosis of major depressive disorder (Depression), 42 with schizophrenia, and 28 with mild cognitive impairment (MCI). The five separate groups are identified as follows: 1) Depression: who have been diagnosed as major depressive disorder and the severity of depression was at least moderate with a recurrent major depressive episode according to Diagnostic and Statistical Manual of Mental Disorders (DSM-IV and DSM-V) criteria. 2) Schizophrenia: who have been diagnosed by the International Classification of Diseases (ICD-10) criteria. 3) MCI and AD group: the cognitive declined participants are constituted of MCI and AD according to the criteria of aging and Alzheimer’s disease [56–57]. 4) Controls: individuals with no neuropsychiatric diagnosis and strict exclusion of neurological conditions, including history of traumatic brain injury or neuroimaging evidence of cerebrovascular diseases. There is a total of 27 subjects with depression, 28 with MCI, 42 with schizophrenia, 96 healthy controls, and 43 with AD. The age range of the participants spans from 18 to 91 years, with the AD and MCI groups specifically ranging from 60 to 87 years [55].

### 2.2 EEG data acquisition and preprocessing

Resting-state EEG was recorded using 19 electrodes configured in accordance with the international 10–20 system. Recordings were conducted at the medical center between 8 am and 1 pm, with a sampling rate of 500 Hz (Nihon Kohden, Japan). Preprocessing included removal of 50 Hz power-line interference, followed by application of a 1 Hz high-pass filter. All pre-processing was performed on the publicly available datasets. Cleaned EEG signals were then bandpass filtered into delta (1–4 Hz), theta (4–8 Hz), alpha (8–13 Hz), beta (13–30 Hz), gamma (30–45 Hz) bands by Butterworth filter.

### 2.3 Functional brain network construction

**Fig 1:**
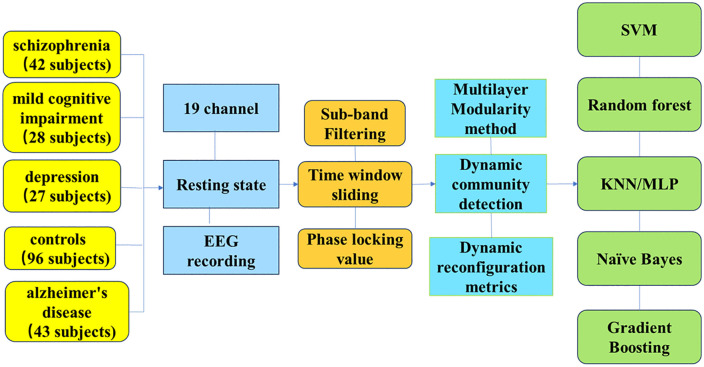
The methodological framework proposed in this study.

Given the time-varying nature of EEG neural oscillations, dynamic functional connectivity analysis is essential for capturing transient network reconfiguration. A non-overlapping sliding window of 2 seconds is implemented to segment the EEG time series within each frequency band. This window length balances temporal resolution with the stationarity assumption required for connectivity estimation [43]. Within each window, frequency-dependent functional networks are generated by the phase locking value (PLV), a robust measure of phase synchronization. Nodes represent EEG recording sites, and edge weights are derived from calculating the instantaneous phase differences between node pairs [58–59]. These PLV measurements then become elements in the connectivity matrix of the brain, which is undirected and weighted. As the non-overlapping sliding window progressed across the entire recording, a sequence of temporal adjacency matrices is generated, forming a multilayer functional brain network for each participant and frequency band. Each layer in the multilayer network corresponds to a discrete time window within the EEG recording. Nodes (EEG channels) are replicated across layers, with intra-layer edges representing instantaneous functional connectivity and inter-layer edges linking the same node across consecutive time points. As illustrated in the proposed analytical framework in Fig 1, these dynamic networks serve as input for subsequent community detection, network reconfiguration analysis and machine learning classification.

### 2.4 Robust community detection

**Fig 2:**
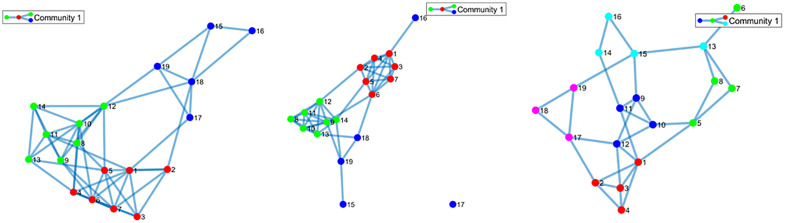
Illustration of a time-varying brain network with evolving community membership. Nodes are color coded by community assignment; identical colors indicate membership in the same community.

In complex networks, communities are defined as clusters of nodes with stronger intra-community connections than inter-community links, thereby facilitating efficient information transfer and functional integration [17,60,61]. As schematically illustrated in Fig 2, nodes in dynamic networks can change their community membership over time. Tracking how communities evolve over time provides critical insights into brain network dynamics [62–65].

To ensure robust community detection in multilayer networks, this study implements a multilayer modularity method comprising of static community detection and null-model-based statistical validation and temporal regularization [64–65]. First, static community detection is performed by the Louvain algorithm within each temporal layer. Maximized modularity quality function is taken to partition the network based on the overall connection strength, when the intra-community edge strength is maximized and the inter-community connections are minimized. And each node is correspondingly assigned to a community membership [64–65].

**Fig 3:**
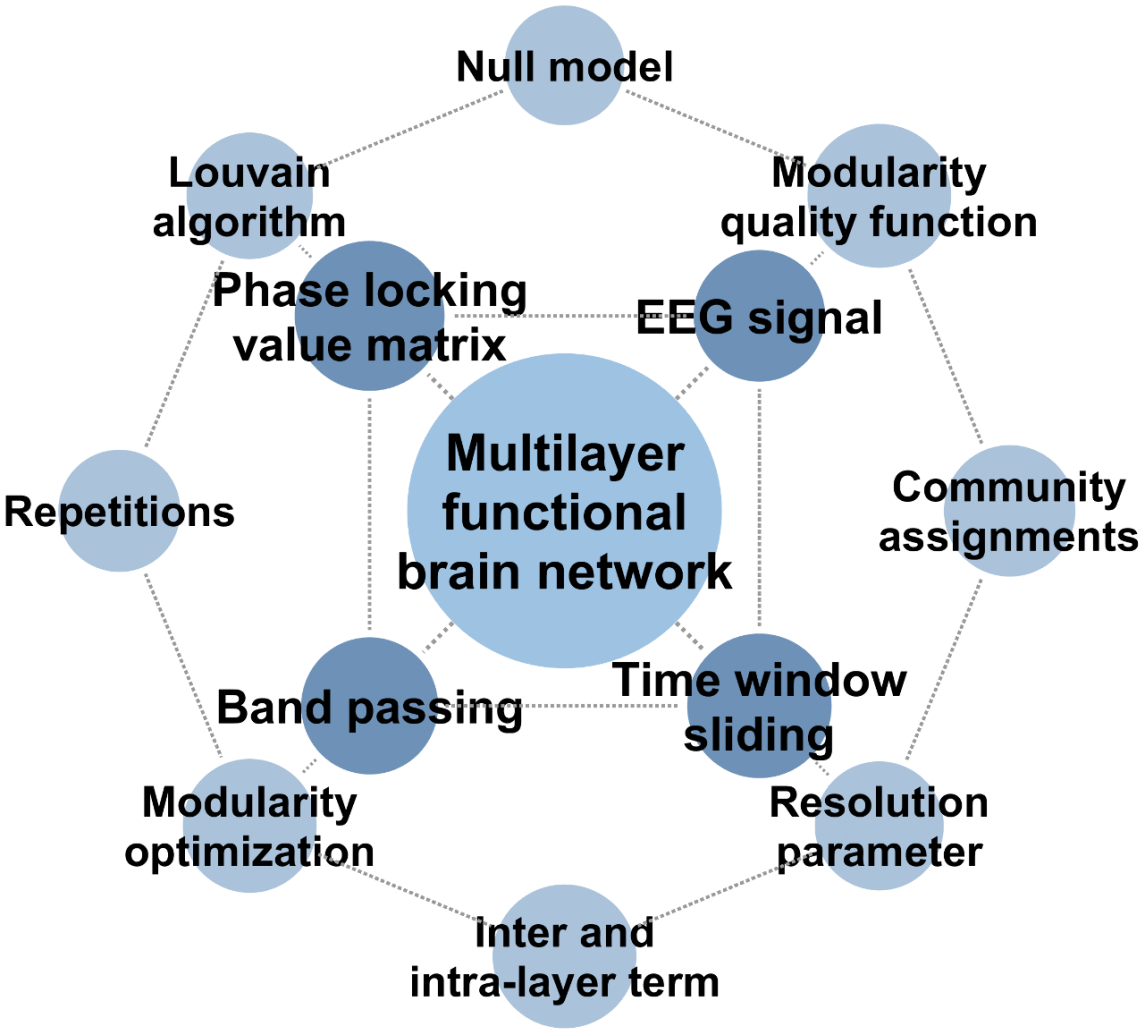
Key steps of the multilayer modularity method for robust community detection.

Statistical null models are then employed to ensure meaningful community detection of the detected partitions, which should be distinguished from stochastic fluctuation as a configuration null model [62,64]. For each layer, one hundred surrogate networks are generated by randomizing edge weights while preserving degree sequences in order to ensure the robustness of the statistical test results. Partitions with modularity significantly exceeding the null distribution are retained (*p* < 0.05, Bonferroni-corrected). Later, temporal consistency is enforced by coupling adjacent layers via an inter-layer similarity matrix and a stability regularized optimization function. A temporal similarity matrix quantifies the node similarity across time points where temporal smoothness is evaluated. Maximized quality ensures the stability of community structure over each time slice. Partition quality and temporal smoothness are then balanced via optimization function, which is performed across 100 random temporal alignment initializations. Fig 3 outlines the key steps of the multilayer modularity method for robust community detection in the study. By integrating statistical null models with modularity optimization, robust community detection is achieved in time-varying brain networks.

### 2.5 Network reconfiguration measurement and classification evaluation

Network reconfiguration arises from communities and nodes as they alter their community memberships over time, which can be quantified in terms of cohesion and disjointedness, ranging from the node level through to the community and global levels. Cohesion refers to the frequency of coordinated behavior, while disjointedness refers to the occurrence of isolated movements at node, community and global levels [62,64–66].

#### 2.5.1 Node cohesion.

Node cohesion quantifies the strength of synchronous behaviors between a node and other nodes within its own community across all network layers. This metric reflects an individual node’s degree of internal tightness within its functional module. A higher value indicates stronger intra-community connectivity and greater functional specialization, suggesting that the node exhibits more exclusively participation in the cognitive processes associated with its assigned community.

#### 2.5.2 Node disjointedness.

Node disjointedness evaluates a node’s tendency to maintain connections with nodes in external communities relative to its intra-community connections across all layers. As a reflection of bridging different functional modules and integrating inter-modular information flow, a higher value indicates better separation from the internal community and more diversified connections across multiple communities.

#### 2.5.3 Community cohesion.

Community cohesion represents the overall density of internal connections among all the recording sites within a specific community across all network layers. This metric assesses how tightly and consistently the electrodes within a functional module are interconnected throughout the multilayer network structure. A higher value signifies better internal fidelity and enhanced self-contained integration capability.

#### 2.5.4 Community disjointedness.

Community disjointedness measures the degree to which a community maintains external connections with other communities across layers. It describes how much a community acts as an independent, segregated unit with inter-community connections. A higher value indicates greater independence and better capability for externally distributed resource allocation to other communities.

#### 2.5.5 Community changes.

Community changes represent the dynamic evolution of the structure of communities within multilayer networks. They measure temporal fluctuations in both the internal coherence and the external connection patterns of communities. As a reflection of multifaceted community reconfiguration comprising community cohesion and community disjointedness, a higher value indicates both stronger internal coordination and external differentiation over time, reflecting better systematic coordination capability.

#### 2.5.6 Cohesion strength.

Cohesion strength reflects how frequently node pairs exhibit synchronous behavior when changing their community memberships. Pairwise node interactions are represented in a cohesion matrix, where the weight of an edge quantifies the incidence of simultaneous community co-transitions between node pairs across temporal layers. Cohesion strength encapsulates the collective behavioral tendencies of nodes, distinguishing between independent reconfigurations and synchronized group dynamics.

Although the per-window cohesion is a static snapshot, its temporal average value equals the zero-order moment of the reconfiguration process: regions that must continuously pay a cost to re-stitch their communities will exhibit persistently lower value, whereas regions with stable intra-module bonds maintain high value. Thus, the value of per-window cohesion serves as an integral measure of how much reconfiguration energy a brain region expends over the entire recording.

Specifically, node cohesion reflects the degree of participation and integration of a given region within its community, based on the strength and closeness of connections between a predefined node (i.e., brain region) and other nodes in the same community. It indicates how frequently nodes joint the same community, while node disjointedness represents the degree of isolated changes of certain node in various communities [64–65]. While node flexibility quantifies the persistence and transition capability of nodes to change community membership over time, high flexibility stands for its sensitivity to external stimulus even in the resting-state.

Community structure reflects network reconfiguration, with cohesive and disconnected switches indicating node movement between communities [62,66]. Community cohesion refers to the density and consistency of connections between nodes across communities, indicating functional synchronization in the global brain. High community cohesion suggests closer collaboration and synchronization. Since the cohesion matrix measures how nodes change collectively across communities, cohesion strength evaluates global cohesion across nodes and communities in the matrix form.

Group differences in reconfiguration metrics were tested using the non-parametric Kruskal-Wallis method, followed by post-hoc multiple comparisons with False Discovery Rate (FDR) correction [67].

Subsequently, six machine learning algorithms are implemented to evaluate the discriminative performance based on the extracted network reconfiguration metrics, including Random Forest (RF), Gradient Boosting (GB), Multilayer Perception (MLP), k-Nearest Neighbors (KNN), Naive Bayes (NB) and Support Vector Machine (SVM) [68]. A repeated random sub-sampling validation scheme is implemented. Each time 20% of the feature data from the target group is randomly selected for cross-validation, the matched data is randomly selected from the remaining feature sets, accuracy, precision, recall and F1 score are quantified to compare classification performance. This randomized selection is repeated 1000 times to ensure robust evaluation.

### 2.6 Data and code availability statement

Data of the EEG recordings are available at: https://orcid.org/0000-0003-3097-4693 and https://datadryad.org/stash/share/AWmC0-Afzx29cOkYDXQ6y2-7HF4GBvG-J-9i8hDQZsw. The code for robust community detection and network reconfiguration analysis is available from the following website: http://commdetect.weebly.com/.

## 3. Results

### 3.1 Significant difference according to network reorganization analysis

**Fig 4:**
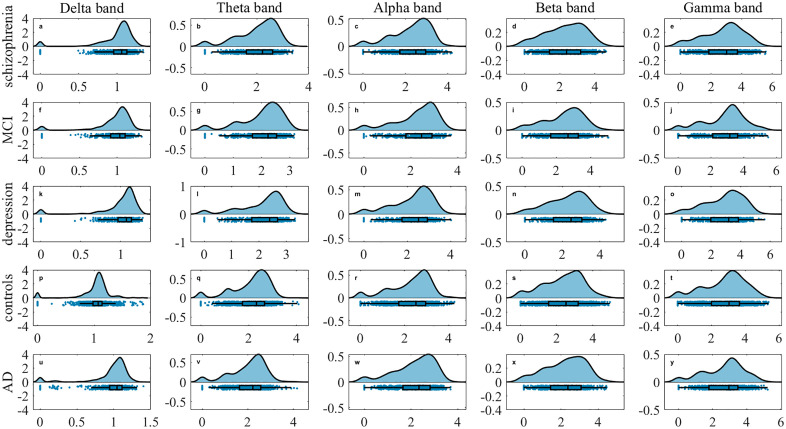
Raincloud plots of community change across five frequency bands. (a-e) schizophrenia, (f-j) MCI, (k-o) MDD, (p-t) healthy controls, (u-y) AD for delta, theta, alpha, beta and gamma bands separately. The y-axis denotes metric magnitude; the x-axis displays the probability density distribution of these values. Individual data points correspond to subject-level measurements.

**Fig 5:**
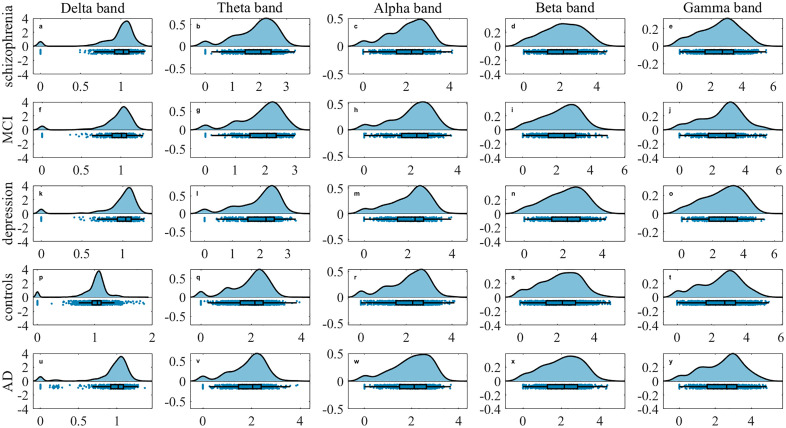
Raincloud plots of community cohesion across five frequency bands: (a-e) schizophrenia, (f-j) MCI, (k-o) MDD, (p-t) healthy controls, (u-y) AD for delta, theta, alpha, beta and gamma bands separately. The y-axis denotes metric magnitude; the x-axis displays the probability density distribution of these values. Individual data points represent subject-level measurements.

**Fig 6:**
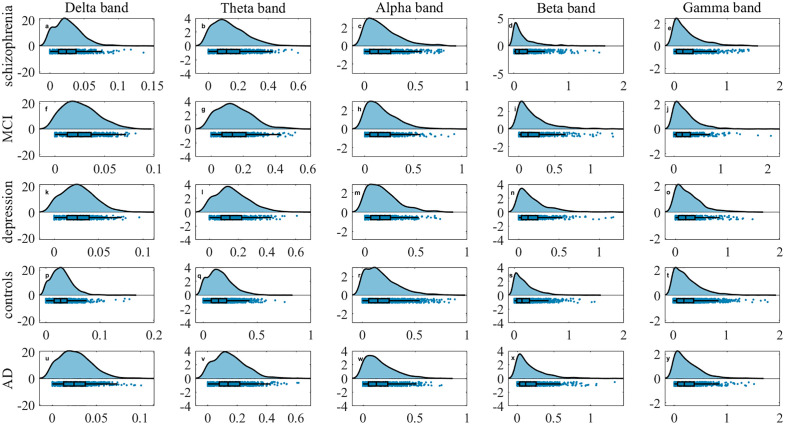
Raincloud plots of community disjointedness across five frequency bands: (a-e) schizophrenia, (f-j) MCI, (k-o) MDD, (p-t) healthy controls, (u-y) AD for delta, theta, alpha, beta and gamma bands separately. The y-axis denotes metric magnitude; the x-axis shows the probability density distribution of these values. Individual points correspond to subject-level measurements.

Robust community detection in multilayer networks is achieved through the proposed framework, which integrates static modularity optimization with statistical validation. Detected partitions are required to exceed the modularity distribution of randomized surrogates (*p* < 0.05, Bonferroni-corrected). Functional brain networks are constructed for each of the five clinical groups across five frequency bands. Application of multilayer community detection and reconfiguration analysis reveal distinct topological signatures. As illustrated in the raincloud plots in Figs 4–6, significant intergroup differences are observed in community changes, community cohesion and community disjointedness metrics separately (corrected *p < 0.001*).

From a global frequency-band perspective across five groups, MDD exhibits the highest community disjointedness in the theta band (*0.16 ± 0.01*), whereas healthy controls show the highest value in gamma (*0.26 ± 0.06*), beta (*0.19 ± 0.05*) and alpha (*0.18 ± 0.03*) bands separately. The MCI group show the greatest community disjointedness in the delta band (*0.19 ± 0.04*). For community cohesion, both MDD (*1.96 ± 0.10*) and healthy controls (*1.96 ± 0.14*) demonstrate elevated value in the theta band. MDD shows a secondary peak in the gamma band (*2.64 ± 0.37*), while healthy controls peaks in the delta band (*0.99 ± 0.10*). The MCI group achieves its highest value in the beta band (*2.26 ± 0.33*). Regarding community changes, MDD demonstrates the highest values in the theta band (*2.12 ± 0.11*) and gamma (*2.89 ± 0.39*) bands. Health controls peak in the delta (*1.02 ± 0.10*) and alpha (*2.27 ± 0.21*) bands, whereas MCI peaks in the beta band (*2.45 ± 0.36*). Globally, healthy controls exhibit the highest community cohesion in the delta and theta band. Both schizophrenia and control groups show significantly higher cohesion in the alpha band globally, whereas MCI and MDD (depression) groups demonstrates substantially elevated cohesion in the beta and gamma bands, respectively.

### 3.2 Topographic and spectral specificity of reconfiguration differences

**Fig 7:**
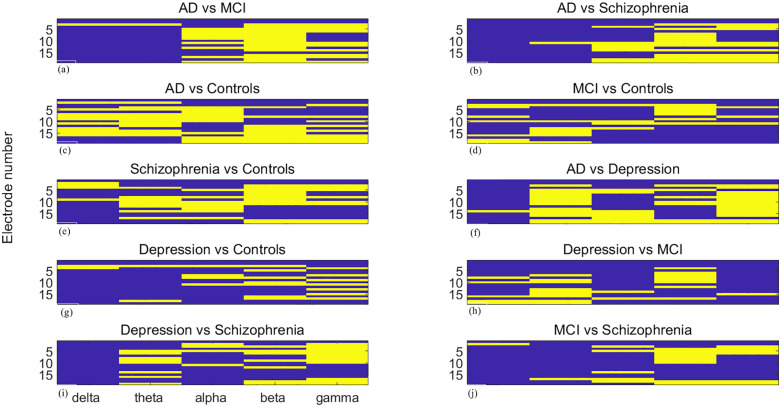
Heatmaps of significant community cohesion differences between paired groups (a) AD vs. MCI, (b) AD vs. schizophrenia, (c) AD vs. controls, (d) MCI vs. controls, (e) schizophrenia vs. controls, (f) AD vs. MDD, (g) MDD vs. controls, (h) MDD vs. MCI, (i) MDD vs. schizophrenia, (j) MCI vs. schizophrenia. The values are across 19 EEG recordings sites and 5 frequency bands. Bright yellow signifies statistical significance (FDR-corrected *p*< 0.05). The x-axis indicates five separate frequency bands, while the y-axis denotes metric magnitude over 19 recording sites.

**Fig 8:**
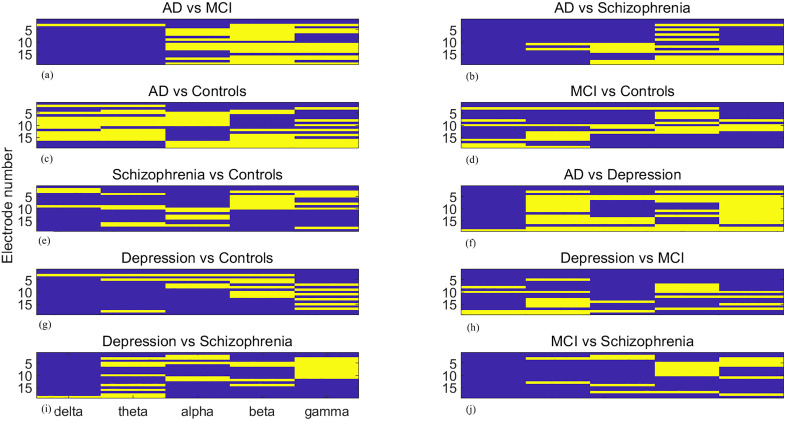
Heatmaps of significant community cohesion differences between paired groups. (a) AD vs. MCI, (b) AD vs. schizophrenia, (c) AD vs. controls, (d) MCI vs. controls, (e) schizophrenia vs. controls, (f) AD vs. MDD, (g) MDD vs. controls, (h) MDD vs. MCI, (i) MDD vs. schizophrenia, (j) MCI vs. schizophrenia. The values are across 19 EEG recordings sites and 5 frequency bands. Bright yellow signifies statistical significance (FDR-corrected *p*  < 0.05)The x-axis indicates five separate frequency bands, while the y-axis denotes metric magnitude over 19 recording sites.

**Fig 9:**
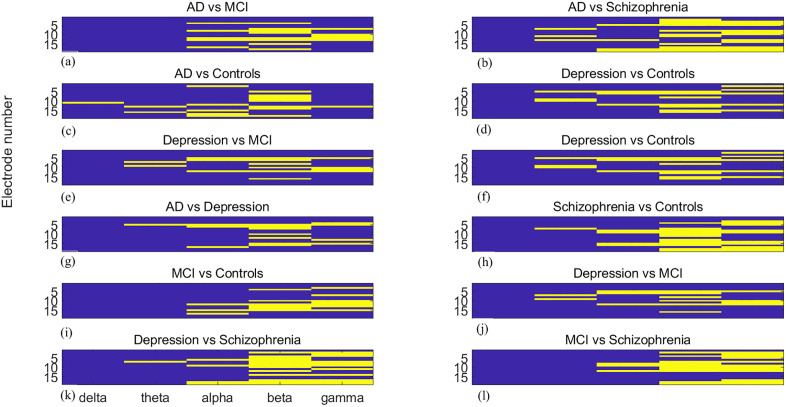
Heatmaps of significant community disjointedness differences between paired groups. (a) AD vs. MCI, (b) AD vs. schizophrenia, (c) AD vs. controls, (d) MCI vs. controls, (e) schizophrenia vs. controls, (f) AD vs. MDD, (g) MDD vs. controls, (h) MDD vs. MCI, (i) MDD vs. schizophrenia, (j) MCI vs. schizophrenia. The values are across 19 EEG recordings sites and 5 frequency bands. Bright yellow signifies statistical significance (FDR-corrected *p* < 0.05).The x-axis indicates five separate frequency bands, while the y-axis denotes metric magnitude over 19 recording sites.

The 19 EEG electrodes, labelled Fp1, Fp2, F7, F3, Fz, F4, F8, T3, C3, Cz, C4, T4, T5, P3, Pz, P4, T6, O1, and O2, are numbered 1 to 19. Brain network reorganization analysis reveals significant topographic and spectral differences between groups. Figs 7–9 present pairwise group differences as heatmaps for community change, community cohesion, and community disjointedness, with bright yellow indicating the existence of significant differences.

Regarding community change metrics, significant differences are observed in the alpha, beta, gamma bands at most recording sites, except for the F7 electrode, which shows significant differences only in the beta and gamma band, when comparing AD with MCI. The delta band illustrates significant differences when comparing AD with healthy controls. The comparison between MCI and controls yields fewer significant differences from the perspective of recording sites, concentrates in delta to gamma bands over frontal regions. When comparing AD and schizophrenia, most significant differences appear in the beta band, with the occipital lobe playing an important role. In comparisons involving depression, most differences are in the alpha band when compared with the AD group, in the gamma band when compared with controls, and in the beta band when compared with MCI, although almost all frequency bands demonstrate significant differences. For depression versus schizophrenia, the frontal and central lobe show significant differences in the gamma band. When schizophrenia is compared with AD, the beta and gamma bands illustrate more significant differences Figs 8,9.

**Fig 10:**
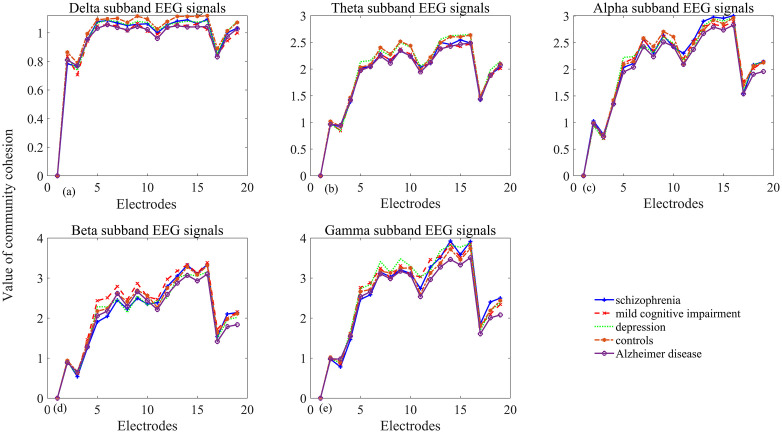
Reduced community cohesion in neuropsychiatric disorders relative to healthy controls across frequency bands. The x-axis represents 19 recording sites, and different line colors and markers in the figure denote the separate groups (a) delta band (b) theta band (c) alpha band (d) beta band (e) gamma band.

Focusing on community changes, as illustrated in Fig 10, significant differences span nearly all recording sites except Fp1. Electrodes F7, F3, Fz, T3, Cz, C4, P3, O1, and O2 are particularly discriminative. The beta band outperforms other bands, accounting for 70% of significant differences at O2. Beta and gamma bands generally surpass lower frequency bands, with beta at Fz, C3, O1, and O2 being especially robust.

Electrode sensitivity varies by clinical conditions. For example, electrodes F7, T3, O1, and O2 are more sensitive to MDD (depression), while F7, F3, T3, and T6 are more sensitive to identifying schizophrenia. In groups with cognitive decline, AD exhibits widespread significance across recording sites and frequency components. In contrast, MCI is best captured by electrodes Cz, T5, F7, P4, T6, O1, and O2. In terms of community disjointedness, schizophrenia exhibits the highest values in the alpha, beta and gamma bands, particularly at the electrodes O1 and O2. The depression group shows the highest value at electrodes C3 and C4. The MCI group demonstrates the highest community disjointedness at electrode F3 in the beta band, while the control group shows the highest value at electrode F7.

### 3.3 Decreased cohesion strength under neuropsychiatric disorders

**Fig 11:**
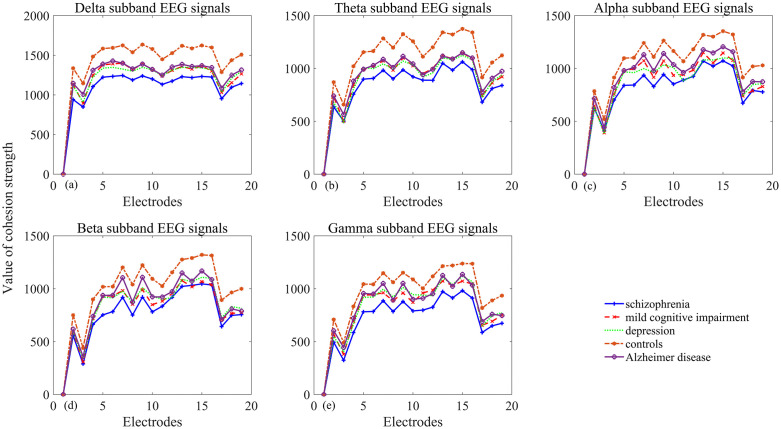
Cohesion strength across groups, sites, and frequency bands. The x-axis represents 19 recording sites, and different line colors and markers in the figure denote the separate groups (a) delta band (b) theta band (c) alpha band (d) beta band (e) gamma band.

Relative to healthy controls, all neuropsychiatric disorder groups demonstrate lower cohesion strength as illustrated in Fig 11. Schizophrenia shows the lowest cohesion strength across all five frequency bands and over all 19 recording locations. AD cohesion exceeds MCI across all five frequency bands, most prominently in beta (F8, C3, T5, Pz) and gamma (F8, C3) bands. MDD (depression) cohesion strength is substantially lower than MCI in alpha, delta and theta bands (notably F4, F8, Cz), but higher in beta and gamma bands.

Taken cohesion strength as a feature, Table 1 reports the classification accuracy via six machine learning algorithms. All pathological group has an accuracy rate above 80%. The results demonstrate that SVM and RF excel for AD and MDD. SVM, RF, and KNN perform better in classifying MCI, while RF and KNN are more effective in classifying schizophrenia.

**Table 1:** Classification accuracy comparison between different classifiers for five-group neuropsychiatric disorder classification (mean* *± std, ‘Schiz’ = schizophrenia).

Group	n	SVM (Gaussian)	Random Forest	Gradient Boosting	Neural Network	Multilayer Perceptron	Naive Bayes
**AD**	*43*	*0.82 ± 0.01*	*0.82 ± 0.01*	*0.78 ± 0.04*	*0.75 ± 0.05*	*0.80 ± 0.02*	*0.39 ± 0.07*
**Controls**	*96*	*0.60 ± 8.88e-16*	*0.67 ± 0.06*	*0.60 ± 0.06*	*0.63 ± 0.07*	*0.65 ± 0.07*	*0.62 ± 0.07*
MDD	*27*	*0.88 ± 0.01*	*0.88 ± 0.01*	*0.85 ± 0.03*	*0.81 ± 0.05*	*0.86 ± 0.03*	*0.57 ± 0.09*
**MCI**	*28*	*0.88 ± 0.01*	*0.88 ± 0.01*	*0.86 ± 0.03*	*0.82 ± 0.05*	*0.88 ± 0.02*	*0.56 ± 0.07*
**Schiz**	*42*	*0.82 ± 0.01*	*0.85 ± 0.03*	*0.84 ± 0.04*	*0.77 ± 0.06*	*0.85 ± 0.03*	*0.81 ± 0.05*
**Mean ACC**	*236*	***0.80 ± 0.01***	***0.82 ± 0.03***	***0.79 ± 0.04***	***0.76 ± 0.06***	***0.81 ± 0.03***	***0.59 ± 0.07***

## 4. Discussion

In this study, resting-state EEG signals are employed to characterize time-varying neural oscillations. Graph theory and multilayer community detection are then integrated to quantify frequency-dependent functional network reconfiguration. This dynamic framework elucidates pathophysiological mechanisms underlying neuropsychiatric disorders, with lower cohesion strength emerging as a robust, transdiagnostic biomarker across all frequency components and recording locations compared to healthy controls.

Most neuropsychiatric disorders represent chronic, debilitating conditions with high morbidity and mortality. Current diagnostic paradigms rely heavily on subjective clinical assessment, lacking objective, scalable and cost-effective tools. The present work addresses this global health crisis by combining multilayer modularity optimization with null model based statistical validation, enabling reliable differentiation of MCI, AD, MDD, schizophrenia, and healthy controls. To our knowledge, this is the first study to leverage dynamic community detection within time-resolved EEG networks to discriminate these pathological conditions. Unlike static connectivity analyses, this approach delves into temporal evolution of modular organization, providing a novel perspective for the diagnosis of neuropsychiatric disorders.

Six machine learning classifiers are employed to assess the discriminative power of these network reconfiguration metrics. Support Vector Machine (SVM), K-Nearest Neighbor (KNN) and Random Forest (RF) classifiers consistently achieve an accuracy rate of over 80% for each pathological group, highlighting the translational potential of these features. A key limitation is the imbalanced sample size, with healthy controls outnumbering clinical cohorts; further validation in larger, balanced datasets is essential. In particular, while cohesion strength proves highly informative, complementary metrics warrant exploration [55].

The brain operates as a modular, adaptive system, wherein functional communities take responsibility for specialized processing while enabling flexible reconfiguration across cognitive demands. Lower cohesion strength reflects the pathological disruption of the intra-modular integration balance. This study demonstrates the diagnostic and classificatory potential of dynamic brain network architecture while laying a theoretical foundation for developing clinical diagnostic tools and neural biomarkers for neuropsychiatric disorders.

In general, complex networks are organized into distinct modules that subserve specialized cognitive functions. Time-varying integration and coordination within these modules are essential for fundamental brain operations, which can be significantly disrupted in pathological conditions. The adaptability of these communities ensures rapid reconfiguration of connectivity patterns across different tasks or cognitive states in response to environmental demands. Alterations in brain oscillations hold substantial diagnostic promise for neurological disorders [69–71]. Schizophrenia primarily affects the prefrontal and cingulate regions, whereas MDD impacts the hippocampus and prefrontal cortex, indicating disorder-specific structural and functional perturbations [72]. In MCI, specific network alterations reflect early neurodegenerative processes associated with cognitive decline, particularly those related to memory and executive function. Functional connectivity has been proposed as an early biomarker for MCI, with significant heterogeneity observed in the dynamic properties of associated brain networks [30]. In AD, reduced global efficiency and increased characteristic path length indicate a shift toward randomized and decentralized brain network topology, as observed in functional magnetic resonance imaging studies [73].

Dynamic network reconfiguration illuminates the brain’s adaptive mechanisms and provides novel evidence for neuropsychiatric research. Enhanced intermodular integration may reflect optimization for task efficiency, whereas diminished community cohesion may indicate inefficiencies in information processing. Lower community cohesion strength may underlie core pathological mechanisms and correlate with specific neuropsychiatric phenotypes. Elucidating cohesive network reconfiguration sheds light on functional degeneration and impaired adaptive processes at the community structural level. For instance, targeted modulation of specific modules may more effectively alleviate pathological conditions. These insights are relevant not only to neuropsychology, but also to neurorehabilitation, cognitive neuroscience and other related fields, facilitating the development of precision intervention strategies.

Although dynamic fluctuations in brain networks across different frequency bands and cognitive tasks have been explored, including delta, theta, alpha, beta and gamma rhythms, their applications to neuropsychiatric disorders remains limited [43]. Given the brain’s inherently time-varying nature, characterizing these dynamic features is critical. Dynamic community structure provides an effective means of tracking evolving modular organizations. Detecting and monitoring community structure is thus essential for uncovering neural dynamics in health and disease. Identifying disrupted dynamic communities facilitates exploration of how network pathology manifests across psychiatric conditions. Simultaneous analysis of multiple connectivity patterns, including resting-state cohesion and disjointedness, across time points and frequency components, enhances understanding of how these changes relate to clinical symptoms and supports the design of targeted interventions, such as electrical stimulation [60,74–76].

Frequency-dependent functional connectivity exhibits systematic and coherent reorganization within and between brain sub-networks. Different frequency bands differentially modulate cognitive functions, understanding their influence on dynamic network reconfiguration is crucial for unravelling complex psychological phenomena. In this study, gamma and delta bands correspond to the highest and the lowest scores, respectively, in specific groups across cohesion, disjointedness and strength metrics. Future research should involve large cohorts and integrate multimodal neuroimaging. Combining community structure analysis with advanced machine learning and deep learning techniques will enable more comprehensive classification. Additionally, the influence of different brain network construction methods on multi-disorder discrimination requires systematic exploration.

## 5. Conclusion

The application of robust community detection and network reconfiguration analysis to routine EEG recordings has proven particularly valuable, uncovering both disorder-specific alterations in community structure and shared pathological mechanisms. This study establishes reduced resting-state network cohesion strength as a consistent, transdiagnostic biomarker of neuropsychiatric disorders. Going beyond traditional static connectivity analyses, which focus on pairwise connections, we demonstrate that community-level reorganization provides deeper mechanistic insights into network pathology. Specifically, weakened community cohesion in the delta band, coupled with reduced community disjointedness in alpha and gamma bands across disorders, reflects fundamental impairments in information integration and segregation at the modular level, offering novel explanations for associated cognitive and functional deficits.

These findings validate EEG-based dynamic network analysis as a clinically valuable tool, and provide a mechanistic framework for understanding how deteriorated dynamic processes contribute to mental and cognitive dysfunction. Future studies should investigate the relationship between community structural trajectories and longitudinal clinical progression, in order to enable personalized intervention strategies. This integrative approach bridges basic network neuroscience and clinical neuropsychiatry, paving the way for objective, scalable diagnostic and therapeutic advances in psychiatry.
